# Comparative study of perinatal outcomes between pregnant women with gestational diabetes mellitus diagnosed by the new IADPSG and the old ADA criteria

**DOI:** 10.1186/1758-5996-7-S1-A83

**Published:** 2015-11-11

**Authors:** Phelipe Guimarães de Ornellas, Luana Verztman Bagdadi, Zuliana Bonde Almeida, Priscila de Almeida Lago, Ana Luíza Campanholo, Joana Rodrigues Dantas, Marcus Miranda dos Santos Oliveira, Melanie Rodacki, Lenita Zajdenverg

**Affiliations:** 1 Universidade Federal do Rio de Janeiro, Rio de Janeiro, Brazil

## Background

In 2010 the International Association of Diabetes and Pregnancy Study Group (IADPSG) suggested new values for the diagnosis of gestational diabetes mellitus (GDM). These values, also accepted in 2013 by the World Health Organization, are lower than those used previously, which resulted in an increase in the number of pregnant women with this diagnosis. It is still controversial if IADPSG/WHO criteria is cost-effective and safe.

## Objective

To compare perinatal outcomes of pregnancies from women with GDM diagnosed in 2004 by the former ADA criteria with those diagnosed in 2014 by the IADPSG criteria /WHO.

## Materials and methods

Data were collected on medical records from pregnant women with GDM who required insulin treatment followed in 2004 and in 2014 at the Maternity School of the Federal University of Rio de Janeiro (UFRJ). The criteria for indicating insulin were the same in both groups: Fasting plasma glucose (FPG) above 95 mg/dL or above 140 mg/dL 1 hour postprandial, 7 days after initiation of diet therapy. It was used SPSS to perform comparative analyzes of the incidence of abortion, hypertensive disorders of pregnancy (HDP), preterm birth, birth weight and weight adequacy for gestational age between the groups.

## Results

GDM diagnosis in 2014 were made only by two abnormal FPG in 36 (50.7%) women and in 20 (28.2%) pregnant women it would not be diagnosed by the old criteria. Women from both yrs. did not have differences on average BMI (29.5 x 28.6 Kg/m2; p=0.10) nor in age (30.3 x 32.0 yrs.; p=0.16). Among the 28 patients analyzed in 2004, 7 (25%) of them delivered a newborn large for gestational age (LGA), while in 2014, the 71 analyzed patients, 3 (4.2%) had LGA infants; p <0.001. The mean weight of newborns in 2004 was 3575 grams, while in 2014 was 3181 grams; p<0.001 There were no statistical differences between abortions, HDP and prematurity rates.

## Conclusion

The findings of this retrospective study in which we compared maternal and fetal endpoints based on two different diagnostic criteria indicates that in women with GDM treated with insulin, intervention is more effective in those diagnosed through the new criteria proposed by IADPSG/WHO. We concluded that diagnose DMG using a higher sensitivity test improves weight parameter of newborns.

